# Sentiment interpretability analysis on Chinese texts employing multi-task and knowledge base

**DOI:** 10.3389/frai.2023.1104064

**Published:** 2024-01-05

**Authors:** Xinyue Quan, Xiang Xie, Yang Liu

**Affiliations:** Beijing Institute of Technology, Beijijng, China

**Keywords:** interpretability analysis, sentiment classification, multi-task training, attention mechanism, knowledge base

## Abstract

With the rapid development of deep learning techniques, the applications have become increasingly widespread in various domains. However, traditional deep learning methods are often referred to as “black box” models with low interpretability of their results, posing challenges for their application in certain critical domains. In this study, we propose a comprehensive method for the interpretability analysis of sentiment models. The proposed method encompasses two main aspects: attention-based analysis and external knowledge integration. First, we train the model within sentiment classification and generation tasks to capture attention scores from multiple perspectives. This multi-angle approach reduces bias and provides a more comprehensive understanding of the underlying sentiment. Second, we incorporate an external knowledge base to improve evidence extraction. By leveraging character scores, we retrieve complete sentiment evidence phrases, addressing the challenge of incomplete evidence extraction in Chinese texts. Experimental results on a sentiment interpretability evaluation dataset demonstrate the effectiveness of our method. We observe a notable increase in accuracy by 1.3%, Macro-F1 by 13%, and MAP by 23%. Overall, our approach offers a robust solution for enhancing the interpretability of sentiment models by combining attention-based analysis and the integration of external knowledge.

## 1 Introduction

Deep learning models have achieved state-of-the-art results in many fields of natural language processing. However, many results cannot be trusted or applied due to the black-box mechanism of deep learning models, especially in medical, military, legal, and other demanding fields. Therefore, how to analyze the interpretability of deep learning models has attracted more and more discussion and attention. Interpretability analysis involves two main aspects: data analysis and model analysis. Data analysis focuses on understanding the data independently before building the model. Common techniques include classification, clustering, and dimensionality reduction. Model analysis can be categorized into intrinsic interpretability analysis and post-modeling interpretability analysis according to time (Du et al., 2019). The former aspect focuses on examining the characteristics of the model itself, such as weights and monotonicity. The latter can be further divided into global interpretability and local interpretability, depending on whether the aim is to explain overall predictions or individual predictions of the model, respectively. In the 2022 Language and Intelligence Challenge, the Chinese Information Processing Society and the China Computer Federation jointly released the sentiment interpretability evaluation task, providing the evaluation indicators and the evaluation datasets. The interpretability analysis in the challenge belongs to global interpretability. The task requires participants to output the sentiment prediction of the model of the text and the evidence on which the prediction depends. Here, the evidence is the tokens in the text that are strongly related to the prediction of the model. We ranked 7th out of the final 48 teams, and in this study, we will discuss the method we used.

When selecting an interpretability analysis method, our preference lies with the attention mechanism. Jain and Wallace (2019) discovered that different attention distributions can yield identical prediction results, leading them to conclude that the attention mechanism is unsuitable as an interpretability method. Wiegreffe and Pinter (2019) proposed four testing approaches to demonstrate that, under specific conditions, the attention mechanism can indeed offer an interpretable foundation for model prediction outcomes. In the context of the sentiment classification task of this study, we contend that sentiment prediction of the model is determined by the output of the hidden layer associated with the classification label, which exhibits strong correlations with other tokens in the text. As a result, we consider the conclusions drawn from employing the attention mechanism as a reliable interpretability basis in this particular scenario.

Based on the granularity of sentiment analysis processing, tasks can be categorized into aspect-level, sentence-level, or document-level analyses (Hemmatian and Sohrabi, 2019). In this study, our focus lies on sentence-level analysis. In data-driven sentiment analysis tasks, it is customary to employ text as the input of the model and predict the sentiment category of the given text as its output. Models are typically trained using annotated training datasets that provide sentiment labels. Various models, such as RNN (Elman, 1990), CNN (Kim, 2014), and BERT (Devlin et al., 2018), can be employed for the sentiment analysis task. In addition to utilizing the classification framework directly, researchers such as Schick and Schütze (2020, 2021) have proposed Pattern Exploiting Training. This approach leverages language models and achieves sentiment classification through generative means by attaching task-specific prompts to the text.

Our approach centers on sentiment interpretability analysis, employing a multi-task training mode in conjunction with an attention mechanism and external knowledge base. Sentiment generation is incorporated as an auxiliary task to facilitate sentiment classification, with the classification task determining the predicted sentiment category. Multi-task training aims to acquire attention scores from multiple perspectives, mitigating potential bias resulting from a single-task attention mechanism. Interpretability analysis methods gauge the contribution of input tokens to model predictions, forming the basis for the selected evidence outputs. Due to vocabulary overflow concerns in word-level models, contemporary classification models primarily adopt a character-based approach. Consequently, the output of evidence may be fragmented, omitting certain portions. Given that Chinese evidence often manifests in phrase form, this issue of phrase splitting significantly affects Chinese sentiment analysis. To address this matter and ensure the retrieval of complete evidence phrases within character-based scoring, we introduce an external Chinese vocabulary as a knowledge base. By comparing against baseline experiments, our method exhibits a notable improvement. To further investigate, we conduct ablation experiments to assess the impact of different modules on the results.

## 2 Related work

In the discussion of interpretability analysis methods, Ribeiro et al. (2016) proposed the local interpretable model-agnostic explanation, which interpreted the local prediction results of complex models by using the linear model to fit the variation in results caused by disturbances to the input instances. Since the method of Ribeiro et al. (2016) cannot handle non-linear features well, it used the linear model to fit the results; besides the analyzed results are independent of each other, it cannot analyze the results of recurrent neural networks well. Guo et al. (2018) proposed the local explanation method using the non-linear approximation based on Ribeiro's method, which approximates the decision boundary of complex models locally by using regression models combined with regularization methods. Springenberg et al. (2014) used the back propagation method to reduce noise in Zeiler's method (Zeiler and Fergus, 2014) that uses the deconvolution method to explain the results of convolutional neural networks. Sundararajan et al. (2016) and Smilkov et al. (2017) delved gradient information to do the complex model interpretation and respectively proposed the integrated gradient and the smooth gradient methods, while Mareček and Rosa (2019) and Pruthi et al. (2019) made full use of attention scores, such as combining syntax trees with attention and generating fake mask attention for model interpretation. Meister et al. (2021) specifically examined how the sparsity of the attention mechanism influences the analysis of model interpretability. Their research delved into the investigation of attention and its impact on interpretability analysis. Agarwal et al. (2021) introduced a local interpretable model-agnostic explanation method that utilized a linear model to interpret the local prediction results of complex models. This approach was shown to be effective in explaining the predictions of different models, such as decision trees, random forests, and neural networks. Multimodal Routing extends the study by Tsai et al. (2020) in local interpretability by providing a framework for locally interpreting the relative importance of different explanatory features to model prediction given different samples. Zhang et al. (2022) proposed a method that uses a linear model to explain complex model predictions. The authors of this study build on this work by proposing a Sentiment Interpretable Logic Tensor Network (SILTN) that enhances interpretability using a differentiable first-order logic language (FOL).

In the domain of interpretability of Chinese texts classification, Liu et al. (2018) used the generated fine-grained information to construct a generative interpretation framework for text classification and proposed explanatory factor for the first time. Liu et al. (2018) also introduced a risk-minimizing training method for the generation-discriminant hybrid model. Yang et al. (2020) proposed a new sentiment analysis model-SLCABG, which is based on the sentiment lexicon and combines Convolutional Neural Network (CNN) and attention-based Bidirectional Gated Recurrent Unit (BiGRU). The scale of the data has reached 100,000 orders of magnitude, which can be widely used in the field of Chinese sentiment analysis. Yan et al. (2021) proposed a sentiment analysis knowledge graph SAKG-BERT model that combines sentiment analysis knowledge and the language representation model BERT. To improve the interpretability of the deep learning algorithm, Yan et al. (2021) construct an SAKG in which triples are injected into sentences as domain knowledge. Li et al. (2022) introduced Shapley value that is inherent in solid theory for factor contribution interpretability to work with deep learning models by taking into account interactions between multiple factors.

## 3 Methodology

**Figure 1:**
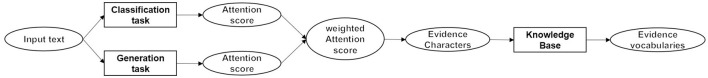
The process of sentiment interpretability analysis.

Our method leverages sentiment datasets. Specifically, we denote the input text of the model as *x*_*i*_ = (*x*_*i*0_, *x*_*i*1_, *x*_*i*2_, …, *x*_*im*_), where *x*_*im*_ represents the *m*th token in the *i*th text, and *x*_*i*0_ represents the < *CLS* > token. The sentiment labels of the text are represented as *y* = (*y*_1_, *y*_2_, …, *y*_*n*_), where *n* denotes the number of texts and *y*_*i*_ ∈ 0, 1. During inference, an interpretability analysis method is employed to provide insights into the prediction of the sentiment category of the model. This study introduces a novel approach that combines a multi-task training mode with an attention mechanism and a knowledge base for interpretability analysis. The multi-task framework includes a sentiment classification task and a sentiment generation task. The knowledge base incorporates the jieba^1^ and idiom vocabularies. The whole process is shown in Figure 1.

### 3.1 Sentiment classification task

The purpose of the sentiment classification task is to predict the sentiment category of the input text, and we implement this task by using a pre-trained BERT (Devlin et al., 2018) model combined with a fully connected network. During training, the output word vector of the BERT (Devlin et al., 2018) model corresponding to the < *CLS* > token can be regarded as the sentence vector representation of the input text and used for the prediction of the fully connected layer. Here, we choose the cross entropy loss function for training, the specific formula is as follows:


(1) $$\begin{array}{l} {\mathrm{loss}}_{1}=-\overset{n}{\underset{i=1}{\sum }}\overset{k}{\underset{j=1}{\sum }}\left(y_{ij}\log_{2}\left(\hat{y_{ij}}\right)+\left(1-y_{ij}\right)\log_{2}\left(1-\hat{y_{ij}}\right)\right) \end{array}$$

where *y*_*ij*_ ∈ {0, 1}, ŷ_*ij*_ ∈ [0, 1] the former represents the true value of the *i*th text on the *j*th sentiment category, and the latter represents the probabilistic prediction value of the model.

### 3.2 Sentiment generation task

The purpose of the sentiment generation task is to predict the sentiment label by generating words of sentiment category. Specifically, it sets appropriate prompt words for the classification task, which are incorporated into the model along with the text to be predicted. The sentiment generation task will be used as an auxiliary task to jointly train the BERT (Devlin et al., 2018) model with the classification task, and the final prediction category of the input text is still obtained by the sentiment classification task.

Given the absence of sentiment word labels in the sentiment dataset, and considering the requirement to generate sentiment category words in the sentiment generation task, we manually formulate the mapping relationship between multiple groups of sentiment words and sentiment categories in a grouping manner and avoid the need for labeling sentiment words by predicting sentiment words within the group; the mapping relationship between emotional words and emotional categories is shown in Table 1.

**Table 1:** Mapping relationship between multiple groups of sentiment words and sentiment categories.

**Positive–negative**
Good–bad; happy–sad; love–hate; joyful–angry; pleased–annoyed; amused–sorrow

Combined with the generation task, the input of the model is extended as: *x*_*i*_ = (< *CLS* >, *x*_*i*1_, *x*_*i*2_, …*x*_*im*_, <*sentiment>*,< *MASK* >), the words that are predicted on the position of < *MASK* > marker are the sentiment words, and they will be the map to the sentiment categories according to Table 1. Furthermore, the prediction of sentiment words is performed separately within each group. The BERT (Devlin et al., 2018) model is pre-trained by mask prediction tasks to obtain a full connection layer with output probability distributed on the vocab. The output vector of < *MASK* > marking position is used to predict emotional words in each mapping group after passing through the full connection layer. Then, the cross-entropy loss function is calculated in each group, and the total loss value of the sentiment generation task is calculated by averaging the sum of losses of each group:


(2) $$\begin{array}{l} {\mathrm{loss}}_{2}=-\frac{1}{G}\underset{G}{\sum }\overset{n}{\underset{i=1}{\sum }}\overset{v}{\underset{j=1}{\sum }}x_{i,j}^{MASK}\left(\log_{2}\left(x_{i,j}^{MÂSK}\right)\right) \end{array}$$

where *G* indicates the number of the groups, as shown in Table 1. For example, Good - bad is a group. The labels of the mapping sentiment categories are positive and negative. *v* is the length of the vocab, $x_{i,j}^{MASK}\in \left\{0,1\right\}$ indicates the true value of the corresponding sentiment words of the < *MASK*>, and the same sentiment category corresponds to different sentiment words in different groups. $\chi_{i,j}^{MÂSK}\in \left[0,1\right]$ indicates the prediction value of the sentiment words corresponding to < *MASK*>. Finally, we train the model with the combination of the sentiment classification task loss and the sentiment generation task loss using multi-task learning. The total loss is as follows:


(3) $$\begin{array}{l} \mathrm{loss}={\mathrm{loss}}_{1}+{\mathrm{loss}}_{2} \end{array}$$

### 3.3 The knowledge base construction

The primary objective of conducting sentiment interpretability analysis is to extract the evidence utilized by the model for sentiment category prediction, specifically focusing on the characters present within the input text. Given the vast number of words involved, the model is susceptible to encountering the out-of-vocabulary (OOV) problem, which motivates the adoption of character-level analysis rather than word-level analysis. By assigning importance scores to individual characters instead of words and assessing their impact on the sentiment category prediction of the model, this approach takes into account the inherent challenges associated with the OOV problem. However, the character-level sentiment analysis will lead to the separation of phrases in Chinese. If the selective output of characters within complete words is considered as evidence, while disregarding the output of complete words, it goes against intuition. To address this issue, we establish a comprehensive knowledge base. When some characters of the input belong to the phrases on the knowledge base and are selected as the evidence, the entire vocabularies can be recalled by the knowledge base and further set a threshold to determine whether the phrases or characters can be the final evidence. We combine the jieba glossary, which is relatively complete and authoritative in Chinese glossaries, with the open Chinese idiom glossary by Tsinghua University^2^ to build the knowledge base. To be specific, first, the phrases longer than three in length in jieba glossary are filtered. Then, the phrases containing letters, numbers, and punctuation symbols are filtered. After that, the phrases with character lengths greater than 4 in the idiom glossary are filtered. Finally, the idioms that can be split into other phrases are filtered. The main purpose of preprocessing the vocab is to reduce the introduction of non-sentiment phrases. For example, the non-sentiment vocabularies combined with the sentiment vocabularies will be filtered in steps 1, 3, and 4. The complete process can be referred to Algorithm 1.

**Algorithm 1:** Vocabulary preprocessing.

1: procedure PreprocessVocab(jiebaGlossary, idiomGlossary)
2: for phrase in jiebaGlossary **do**
3: if WordLength(phrase) >3 **then**
4: if ContainsSymbols(phrase) **then**
5: FilterOut phrase
6: end **if**
7: end **if**
8: end **for**
9: for phrase in idiomGlossary **do**
10: if WordLength(phrase) >4 **then**
11: if CanBeSplit(phrase) **then**
12: FilterOut phrase
13: end **if**
14: end **if**
15: end **for**
16: end **procedure**
17: function WordLength(phrase)
18: return length of phrase
19: end **function**
20: function ContainsSymbols(phrase)
21: return True if phrase contains letters, numbers, or punctuation symbols
22: end **function**
23: function CanBeSplit(phrase)
24: return True if the idiom can be split into other phrases
25: end **function**

### 3.4 Importance score based on attention-mechanism

The fundamental concept underlying the BERT (Devlin et al., 2018) model is the utilization of a multi-head attention mechanism, wherein the attention score is computed as follows:


(4) $$\begin{array}{l} \begin{array}{l} Q={\text{W}}_{\text{q}}\text{X}\\ K={\text{W}}_{\text{k}}\text{X} \end{array} \end{array}$$

(5) $$\begin{array}{l} \begin{array}{l} \text{score}=\mathrm{softmax}\left(\frac{\text{Q}*{\text{K}}^{\text{T}}}{\sqrt{{\text{d}}_{\text{k}}}}\right) \end{array} \end{array}$$

Let X denote the word embedding vector associated with the input text, while *W*_*q*_ and *W*_*k*_ represent the learnable variables, and *d*_*k*_ represents the dimension of vector *K*. The attention score assigned to each word captures the relative significance of the surrounding words with respect to it, thereby enabling the attention score to serve as a metric for evaluating the contribution of the input text to the prediction of the model. Consequently, the attention score ranking can be leveraged to generate the evidence, supporting the prediction of the model. This study is trained with a combination of classification and generation task, the former is realized with the help of < *CLS* > marks, and the latter with < *MASK* > marks, so we will calculate the attention score used to measure the importance of characters by calculating the weighted sum. The specific calculation formula is as follows:


(6) $$\begin{array}{l} score=\alpha *\underset{L}{\sum }{\text{score}}_{<\text{CLS}>}+\left(1-\alpha \right)*\underset{L}{\sum }{\text{score}}_{<\text{MASK}>},\alpha \in \left[0,1\right] \end{array}$$

where α is the task weight, *L* is the number of layers, *score*_<*CLS*>_, and *score*_<*MASK*>_, respectively, calculating the attention scores at the corresponding positions of the subscripts. The model is made up of 12 sub-layers and attention score of each layer will be calculated; therefore, we regard the attention score of each sub-layer corresponding to the position as the attention score value of the model at the corresponding position, and the value of the final attention score at the corresponding character position of the input text is regarded as its contribution value to the model prediction.

### 3.5 Output of the evidence

The scores for each character in the input text are calculated in Section 3.4, denoted as *S*(*x*_*i*_, *s*_*i*_), where $s_{i}=\left(s_{i}^{1},s_{i}^{2},\ldots s_{i}^{m}\right)$. Here, $s_{i}^{m}$ represents the score corresponding to the *m*th character ($x_{i}^{m}$) in the *i*th input text. A higher score indicates a greater contribution of $x_{i}^{m}$ toward predicting the sentiment category *y*_*i*_. The characters in the input text are sorted in descending order based on their scores. The top l*a characters are selected, where *l* is the length of the input text and *a* is the proportion of evidence in the text (determined during data preprocessing). If the selected characters form a phrase that matches the knowledge base constructed in Section 3.3, the phrase is considered as evidence. If the selected characters do not form a phrase or the phrase is not present in the knowledge base, the individual characters themselves are considered as evidence. Finally, the evidence is outputted based on its position in the original text.

## 4 Experiments

We utilize the BERT-base^3^ model with a hidden size of 768 on an RTX 3080Ti. Our training setup includes a batch size of 48, a maximum sequence length of 128, a learning rate of 1*e*−4, and a training duration of 3 epochs. Additionally, we set the parameter α to 0.5, achieve an accuracy of 30.10%, and utilize the AdamW optimizer from Huggingface Transformers.

### 4.1 Datasets processing

The evaluation dataset used in the experiment is the A-list dataset of the emotionally interpretable track in the 2022 Language and Intelligence Technology Competition, with 1,044 data. This competition did not provide the training dataset, so we used the sentence-level sentiment classification dataset from ChnSentiCorp as the training set, with 12,000 data, and the sentiment labels are positive and negative. Since we set the maximum length of input text to 256, part of the data in the training dataset will be truncated to affect the sentiment category. First, we train the model with the training dataset and then delete the data whose predict label and the true label do not match. After that, the length of the dataset is reduced to 11,478. There are distribution differences between the evaluation dataset and the training dataset, and to make full use of the evaluation dataset to reduce its impact on the experimental results, we use the sentiment classification task, the sentiment generation task and the natural language inference task to train the BERT (Devlin et al., 2018) model with the training dataset and then apply the model to the prediction of the evaluation dataset. We use the voting mechanism to label the evaluation dataset by predicting the results and then merge it into the training dataset for training.

### 4.2 Metrics

The metric used in this study is the same as that of the competition, and the evaluation is carried out from three dimensions: the accuracy of model classification, the rationality, and loyalty of the prediction-dependent evidence. The rationality of evidence-dependent predictions is calculated by *Macro*−*F*1 values, to assess the coincidence degree between the evidence predicted by the model and the manually labeled evidence, which is calculated as follows:


(7) $$\begin{array}{l} \mathrm{Macro}-F1=\frac{1}{\text{N}}\overset{\text{N}}{\underset{\text{i}=1}{\sum }}\left(2*\frac{{\text{P}}_{\text{i}}*{\text{R}}_{\text{i}}}{{\text{P}}_{\text{i}}+{\text{R}}_{\text{i}}}\right) \end{array}$$

(8) $$\begin{array}{l} P_{i}=\frac{\left|s_{i}^{p}\cap s_{i}^{g}\right|}{\left|s_{i}^{p}\right|},R_{i}=\frac{\left|s_{i}^{p}\cap s_{i}^{g}\right|}{\left|s_{i}^{g}\right|} \end{array}$$

where $s_{i}^{p}$ and $s_{i}^{g}$ represent the dependent evidence of *i*th data predictions of the model obtained by interpretable analysis methods and the dependent evidence on manually sentiment labeling of *i*th data. The loyalty of evidence-dependent prediction is to evaluate the consistency of the model on the original input evidence and disturbance input evidence prediction by calculating the Mean Average Precision (MAP) of all categories, the formula is as follows:


(9) $$\begin{array}{l} \text{MAP}=\frac{\sum_{i=1}^{\left|X^{a}\right|}\left(\sum_{j=1}^{i}F\left(x_{j}^{a},x_{1:i}^{o}\right)\right)/i}{\left|X^{a}\right|} \end{array}$$

where *X*^*o*^ and *X*^*a*^ represent the order of importance of characters in the original input and perturbed input, respectively. The importance of characters in this article is measured by attention score. |*X*^*a*^| represents the number of characters in the perturbation input, and $x_{1:j}^{0}$ represents the top *j* most important characters in the perturbation input. Functions *F*(*x, Y*) are used to determine whether the character *x* is contained in list *Y*, and if so, *F*(*x, Y*) = 1. A higher MAP value indicates that the model is more consistent on the evidence prediction before and after the perturbation is added.

### 4.3 Baseline

In this study, the interpretability analysis is based on the attention mechanism, and the integral gradient and the linear model are selected as the baseline. To reduce the impact of the framework of model selection on the interpretable prediction, we selected the BERT (Devlin et al., 2018) model as the basic model framework of the baseline experiment.

1) Attention-based interpretability analysis method: The choice of interpretability method in this study is essentially based on attention mechanism. It calculates the attention score of classification marks on other characters to obtain the dependence on input characters in the prediction task. The hidden output vector of classification marks is related to the connection of other characters through attention mechanism strongly. As a result, attention mechanism is credible as an interpretability analysis method.

2) Gradient-based interpretability analysis method: Sundararajan et al. defined interpretability analysis as attribution calculation, that is, to calculate the attribution vector of the input vector relative to the baseline vector. The value of the position corresponding to the input vector in the attribution vector can be regarded as the contribution of the position character to the predicted value. The attribution vector is obtained by integrating the derivative of the input vector on the neural network between the baseline vector and the input vector.

3) Linear-based interpretability analysis method: Ribeiro et al. realized the partial interpretability analysis of the target model by training simple interpretable linear models to fit the partial results of complex target models. The training dataset of interpretable models consists of input data and disturbance data that add disturbance terms to the input data. The label of disturbance data is obtained from the prediction of the target model. In this experiment, we use the ridge regression model as an interpretable linear model.

### 4.4 Results and discussion

The experimental results are shown in the Table 2. The higher the accuracy, *Macro*−*F*1 and *MAP* values, the better the model performances. The highest values of each index are marked in bold. The results in the table show that our method is superior to the baseline model in three indicators. Compared with the *Macro*−*F*1 indicator and *MAP* indicator of Attention-based and Gradient-based, a high Macro-F1 indicator does not mean a high *MAP* value. Analyzing from the calculation formula of indicators (*Macro*−*F*1-formula 7, *MAP*-formula 9), the former represents the accuracy of evidence prediction and directly reflects the effectiveness of interpretable methods. The latter represents the consistency of the evidence prediction before and after the disturbance term is added to the input data, which, to some extent, represents the robustness of the interpretable method. If the complete input text and disturbance text are output as prediction evidence instead of choosing evidence by the proportion, a higher *MAP* value and a lower *Macro*−*F*1 value can be obtained. On the contrary, if the length of output evidence is kept as short as possible, a relatively higher Macro-F1 value and a lower MAP value can be obtained. If the correctness and completeness of output evidence cannot be guaranteed, it can be based on the use scenario to properly adjust the proportion of evidence in the text, to balance the effectiveness and robustness of the interpretable method.

**Table 2:** Experimental results on the competition dataset.

**Method**	**Accuracy**	**Macro-F1**	**MAP**
Attention-based	0.88985	0.51472	0.43279
Gradient-based	0.85057	0.49694	0.43770
Linear-based	0.87835	0.43340	0.39002
Ours	**0.90230**	**0.64406**	**0.66278**

The bold values mean that they are the best result of certain experiment.

### 4.5 Ablation experiments

Compared with the interpretable method based on attention mechanism, our method integrates the pre-processing of evaluation dataset, multi-task training, and evidence recall based on external knowledge base. We analyzed the effects of each module through ablation experiments. The ablation experiments results are shown in Table 3. The definition of methods in the table is as follows: *Ours*−*P* means to remove the pre-processing module of evaluation data, that is, do not automatically label the evaluation data but retain the cleaning of training dataset; *Ours*−*C* means to remove the sentiment classification task, and the sentiment generation task is used for model training; *Ours*−*G* means to remove the task of sentiment generation, and model training is conducted by sentiment classification task; *Ours*−*K* means to remove the external knowledge base, and the evidence output of the model is only determined by the set of evidence proportion and importance score.

**Table 3:** Results of ablation study on the competition dataset.

**Method**	**Accuracy**	**Macro-F1**	**MAP**
Ours-P	0.89464	0.56777	0.61602
Ours-C	0.89847	0.61973	0.65888
Ours-G	0.89963	0.61849	0.66807
Ours-K	0.89751	0.56930	0.48746
Ours	**0.90230**	**0.64406**	**0.66278**

The bold values mean that they are the best result of certain experiment.

Based on the analysis of the ablation experiment results, the accuracy is basically not affected by the removal modules. We maintain that the accuracy of sentiment classification is mainly determined by the model structure and training dataset, which means that the training set selected in this study and the pre-trained BERT (Devlin et al., 2018) model are sufficient to achieve a high accuracy of sentiment prediction. Compared with *Macro*−*F*1 indicators, we believe that the multi-task architecture training model can mitigate the bias caused by single task by calculating the weighted sum of attention of different tasks, thus improving the accuracy of evidence prediction. The pre-processing of the evaluation dataset can automatically label the evaluation data through the model so that the evaluation data can be incorporated into the model training process. The accuracy of the evidence prediction of the model trained by the evaluation data can be improved to a certain extent. The external knowledge base can take the complete recall of the split phrase as evidence, which can be applicable to the circumstances that the evidence phrase is split and taken out as evidence. At the same time, the external knowledge base will introduce the error caused by the recall of the non-evidence phrase which is of great importance for the selection of the knowledge base. In the evaluation dataset selected in this study, the external knowledge base can improve the rationality of evidence prediction. Moreover, in some cirmustances, the knowledge base can be constructed manually.

Compared with *MAP* indicators, the external knowledge base will greatly affect the loyalty of the model. Its analysis is described above; the introduction of the knowledge base will improve the anti-interference of the model in the evidence extraction process by completing the evidence characters. Compared with the knowledge base module, other modules have a relatively weak impact on loyalty of the whole model. We consider that this is also due to the fact that BERT (Devlin et al., 2018) model is sufficient to achieve the task of sentiment classification. Therefore, on the premise of more accurate output of evidence, the combination of knowledge base can enhance the anti-interference of the model.

### 4.6 Case study

**Figure 2:**
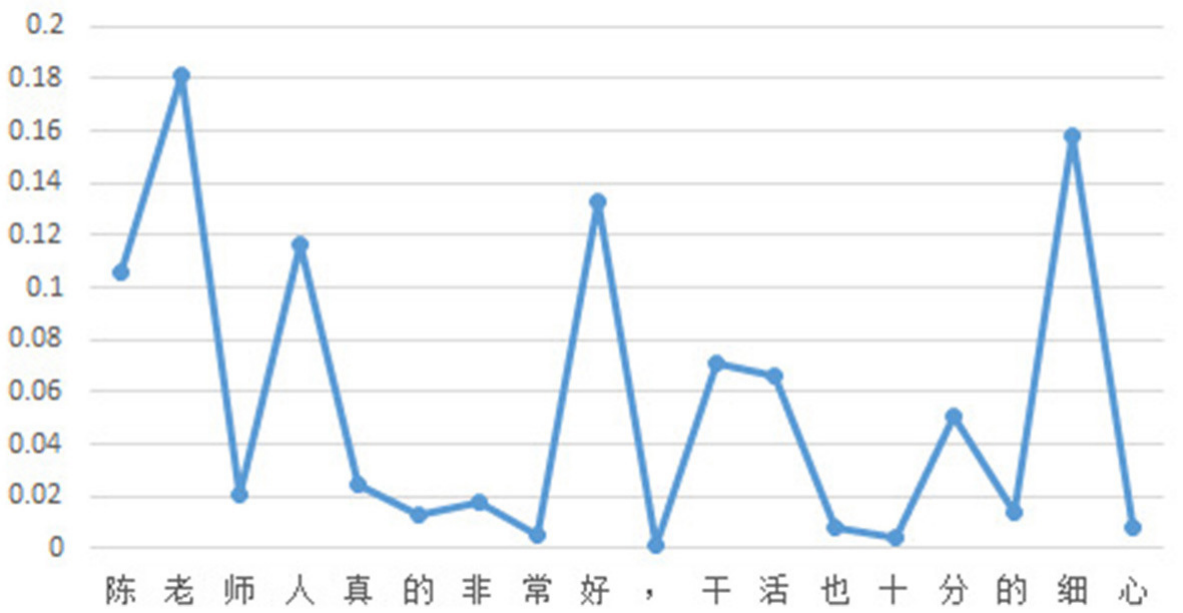
Attention scores of classification task.

**Figure 3:**
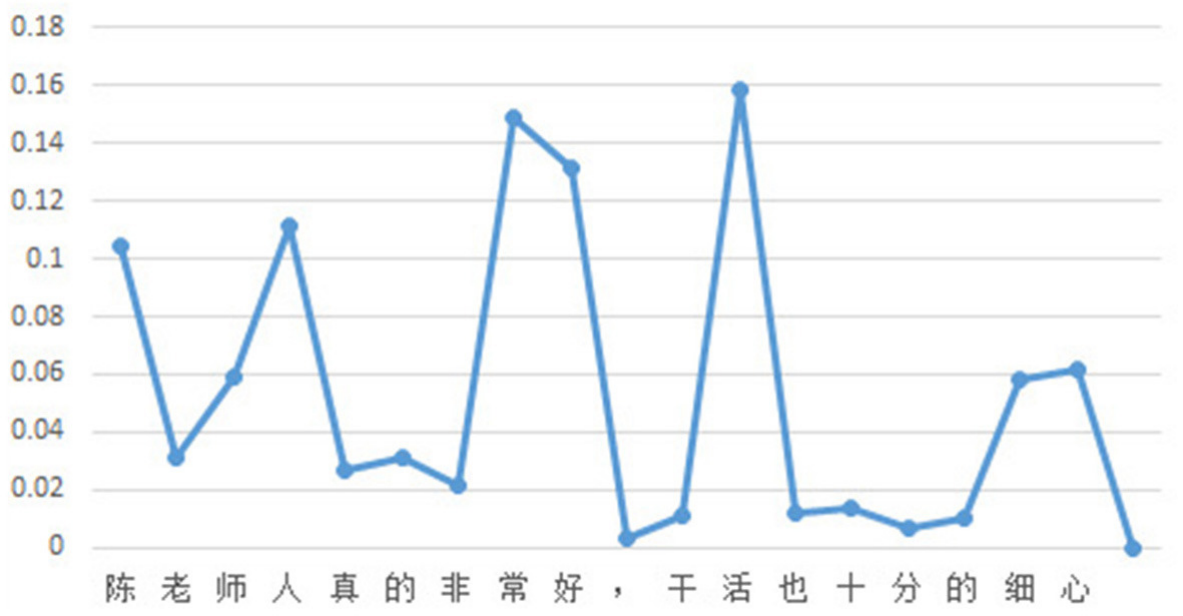
Attention scores of generation task.

To facilitate readers' understanding of the proposed method for sentiment interpretability in this study, a simple example is provided. Given the input text “陈老师人真的非常好,干活也十分的细心” (which translates to “Teacher Chen is very diligent and does work with great attention to detail"), as shown in Figure 2, the attention scores for each character in the classification model are displayed. Similarly, Figure 3 shows the attention scores for each character in the generation model. The magnitude of the attention scores reflects the contribution of each character to the sentiment prediction of the input text. In other words, characters with higher attention scores are more likely to serve as evidence for sentiment prediction.

**Figure 4:**
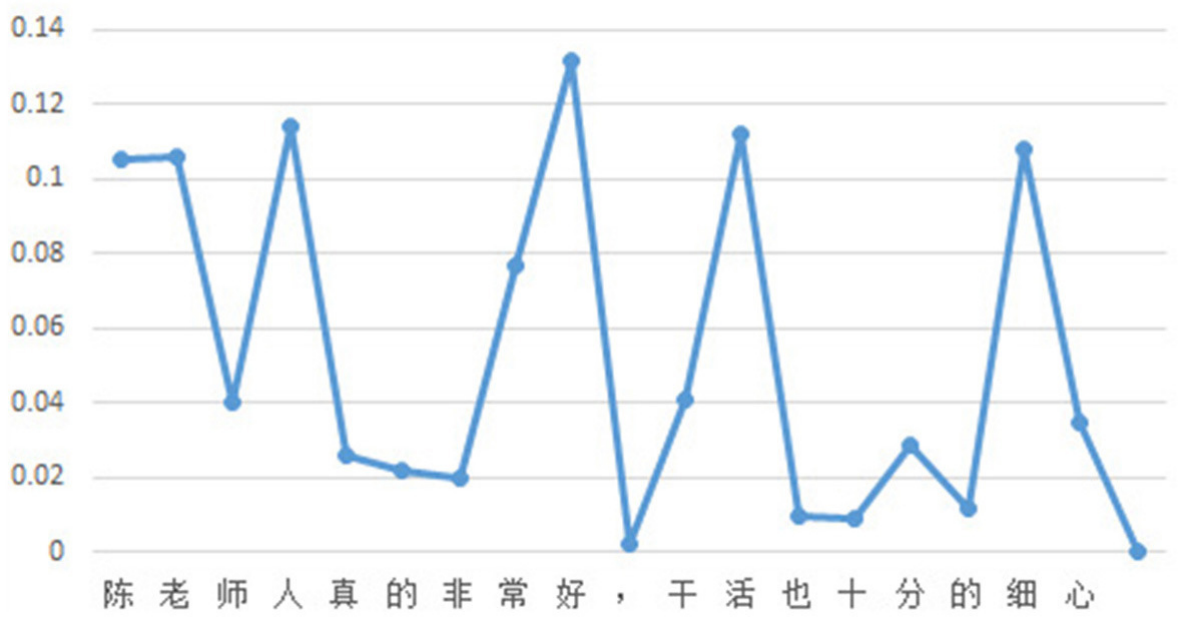
Weighted attention scores.

**Figure 5:**
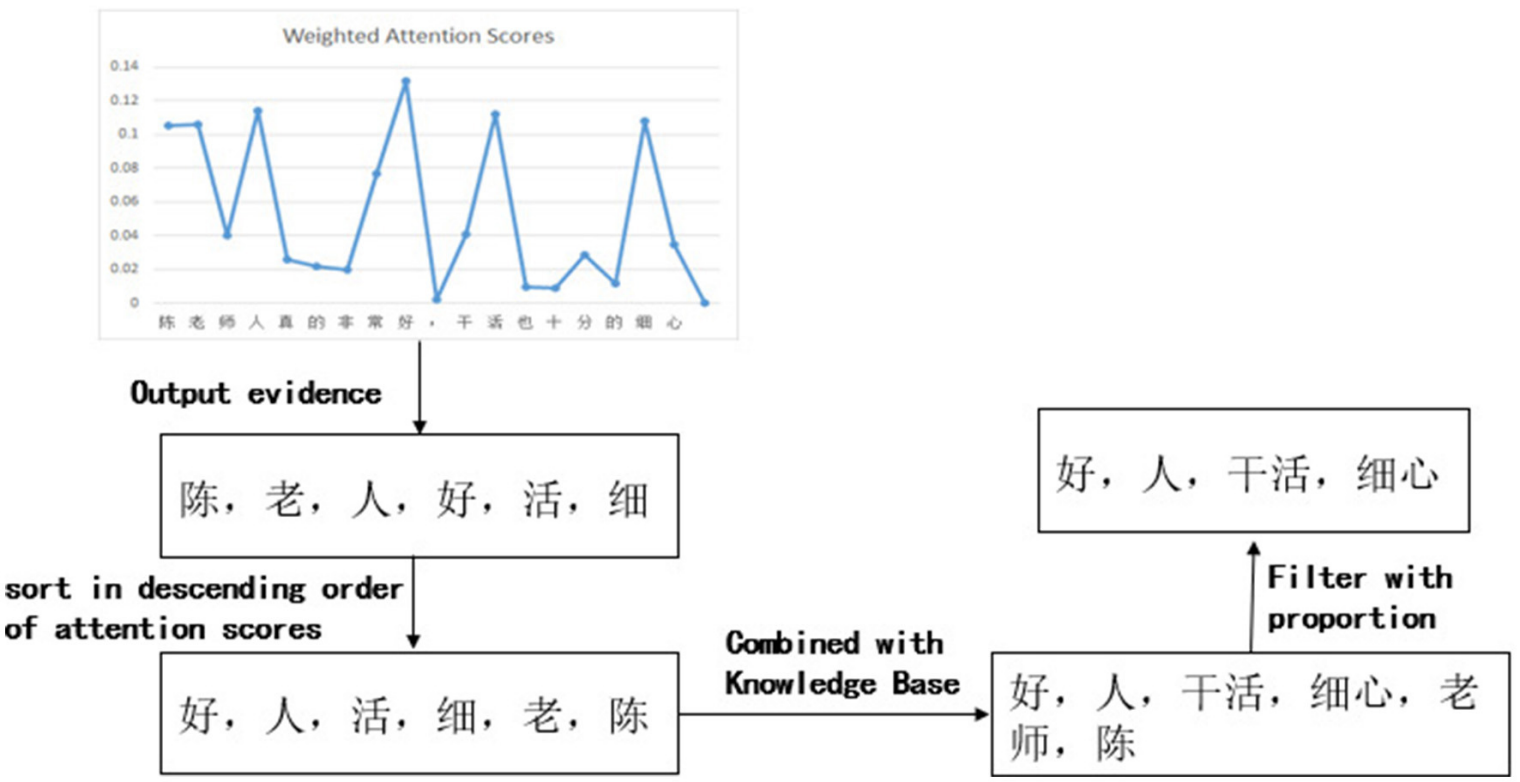
The process of evidence output.

In this case, the weighted attention scores of the classification model and the generation model, with a selected value of 0.5 for parameter “*a*,” are shown in Figure 4. The entire process of evidence word output is presented in Figure 5. It can be observed that the relative magnitudes of the weighted attention scores play a significant role. The characters “陈” (Chen, a given name in Chinese), “ 老” (old), “人” (person), “好” (good), “活” (live), and “细” (thin) have relatively high attention scores. After sorting them in descending order, the sequence “ 好” (good), “人” (person), “活” (live), “细” (patience), “老” (old), and “陈” (Chen, a given name in Chinese) is obtained. By combining these characters with the phrases from the knowledge base, individual characters are connected to form complete words. In this way, some meaningless characters can become evidence vocabularies in the knowledge base. Finally, by applying a proportional value, the ultimate evidence for predicting sentiment is determined as “好,” “人,” “干活,” and “细心,” which are “good,” “person,” “working," and “patience” in English.

## 5 Conclusion

To address the challenge of interpreting sentiment analysis, this study proposes a language model trained using a multi-task learning approach, augmenting with an attention mechanism to assign scores to evidence. Furthermore, an external knowledge base is employed to retrieve complete evidence phrases, thus enhancing prediction rationality and loyalty of the model. Through evaluating and analyzing the competition dataset, it is observed that Chinese dependent evidences, typically in the form of phrases, greatly benefit from the utilization of an external knowledge base. This enhancement is evident in both the loyalty index, measured by the Mean Average Precision (*MAP*) value, and, to some extent, the rationality index, denoted as the *Macro*−*F*1 value. Notably, the multi-task architecture primarily influences the *Macro*−*F*1 value. Moreover, experimental analysis reveals that the manually set evidence proportion and the selection of the knowledge base significantly influence the results. Hence, striking a balance between the rationality and fidelity of evidence extraction tasks by appropriately configuring the evidence proportion and knowledge base selection represents a crucial area for future research endeavors.

## Data availability statement

Publicly available datasets were analyzed in this study. This data can be found at: https://aistudio.baidu.com/aistudio/competition/detail/159/0/leaderboard.

## Author contributions

XQ leads in the completion of experiments and writes the paper. XX guides the direction and goal of the experiment from a global perspective, and guides the revision of the paper at the same time. YL proposes the method of the paper and grasps the content of the paper. All authors contributed to the article and approved the submitted version.

## Footnotes

^1^Jieba is a popular Chinese text segmentation library commonly used in natural language processing tasks. It provides efficient and reliable word segmentation for Chinese text, enabling various applications such as text analysis, information retrieval, and machine translation. Developed by Sun Junyi, Jieba utilizes a combination of different segmentation algorithms to achieve accurate word boundary identification. The library has gained significant popularity within the Chinese NLP community due to its ease of use, flexibility, and excellent performance.

^2^Tsinghua University's Chinese Idiom Glossary is a valuable resource developed by experts at Tsinghua University, providing detailed explanations and interpretations of Chinese idioms. It serves as a comprehensive reference for learners, researchers, and enthusiasts of Chinese language and culture, offering insights into the meanings, usage, and cultural significance of a wide range of idiomatic expressions. The accuracy, reliability, and scholarly approach of the glossary make it an indispensable tool for anyone interested in exploring the rich linguistic and cultural heritage of Chinese idioms.

^3^The model used in this study is an open-source model from Hugging Face, available at: https://huggingface.co/bert-base-chinese.
